# In Trust We Trust: Epistemic Vigilance and Responsibility

**DOI:** 10.1080/02691728.2022.2042420

**Published:** 2022-02-28

**Authors:** Neil Levy

**Affiliations:** a Department of Philosophy, Macquarie University, Sydney, NSW, Australia; b Uehiro Centre for Practical Ethics, University of Oxford, Oxford, UK

**Keywords:** Trust, testimony, epistemic injustice

## Abstract

Much of what we know we know through testimony, and knowing on the basis of testimony requires some degree of trust in speakers. Trust is therefore very valuable. But in trusting, we expose ourselves to risks of harm and betrayal. It is therefore important to trust well. In this paper, I discuss two recent cases of the betrayal of trust in (broadly) academic contexts: one involving hoax submissions to journals, the other faking an identity on social media. I consider whether these betrayals suggest that we ought to be less trusting in contexts like these. I argue that we should not: the acquisition of knowledge is dependent on trust, and we cannot intentionally reduce the extent to which we trust in these kinds of contexts without risking destroying it utterly. Instead, we must trust in our epistemic networks and the way they work to filter out deception.

Trust makes us vulnerable. When we trust someone, we rely on her. We depend on her to keep our secrets or to look after our interests; in so doing we expose ourselves to risks of harm, sometimes small (for example, when I trust someone to pick up the milk on her way home), sometimes very large (for example, when I trust someone with my deepest secrets). Anytime we rely on someone or something, we expose ourselves to risks of harm, but trust goes beyond reliance in ways that make the potential for harm much greater. It’s controversial what trust involves in addition to reliance, but uncontroversial that trust is deeper. It involves putting any doubts about the trustee aside. It thereby exposes us to greater risks but also to qualitatively different kinds of risks: only when we are let down by those we trusted do we feel reactive attitudes like betrayal (Holton 1994; Hawley 2014). Betrayals of trust hurt, often deeply.

In this paper, I will focus on two recent and very different instances of betrayal of trust in academic contexts; one in the peer review process, the other in the much less formal context of social media. These two contexts are obviously extremely different in multiple ways (the first is, among other things, a barrier to entry; the second is accessible to almost everyone in academia; the first is a venerable institution, in which norms have evolved over centuries; the second is novel and norms remain unsettled; and so on). However, I will suggest that they have an important commonality: they are both heavily reliant on trust to play the roles for which we value them. The betrayals of trust in these contexts therefore threaten these goods. These betrayals of trust reveal different vulnerabilities in academic life. I will argue, however, that we should resist the temptation to trust less in these contexts. Those who were deceived behaved appropriately; they satisfied the demands of epistemic responsibility. While these incidents are unusual, and arise due to features specific to the contexts in which they occurred, they may have lessons for other contexts, inside and beyond academia. Agents embedded in well-functioning epistemic networks should adopt an attitude of default trust, only questioning the basic sincerity of claims when they become aware of potential deception.

In Section 1 of this paper, I will present two cases in which trust was betrayed in a (broadly) academic context. In Section 2, I’ll sketch the case for the epistemic value of trust, as crucial to the acquisition of significant truths. Section 3 briefly sets out the nature of trust, before asking how trust can be compatible with epistemic vigilance. Section 4 examines the prospects for increasing our implicit or our explicit vigilance. I argue that any attempt to reconfigure our epistemic vigilance so we are less trusting is likely to damage trust quite extensively. I also argue that such efforts risk worsening the testimonial injustice to which many speakers are already exposed. I conclude that we ought to continue to adopt the attitude of default trust in the contexts I’m concerned with, unless and until fraud becomes so pervasive that the harms of default trust begin to outweigh its epistemic benefits.

## Trust betrayed: Two cases

In 2017–18, three authors wrote a series of hoax articles and submitted them to a range of journals in the fields they call ‘grievance studies’; fields like gender studies and critical race theory in which work is focused on the ways in which members of certain groups are taken to suffer harms and discrimination in virtue of their identities. They aimed to show that these journals would accept for publication ‘some little bit of lunacy or depravity’ (Pluckrose, Lindsay, and Boghossian 2018). The authors maintain that ‘grievance studies’ are in the grip of postmodern relativism, combined with a commitment to certain narrow ideological views: this combination leaves journals in these areas willing to publish weak or even meaningless work, so long as it hews to the party line.

The authors intended their ‘experiment’ to run into 2019, but were forced to bring it to a premature close when the journal *Gender, Place, and Culture* and reporters for news outlets attempted to confirm the identity of the purported author of one article. By that point, 7 of the 20 papers submitted had been accepted and 6 rejected; the rest remained under submission.^1^ The papers accepted ranged in plausibility, as well as theme: accepted papers argued, among other things, that dogs engage in rape culture, that there should be a category of fat bodybuilding in the sport, and that men could reduce their transphobia by anally penetrating themselves with sex toys. At least one paper was widely seen as sufficiently ludicrous by (mainly conservative) news sources to trigger an investigation into the identity of the purported author. To that extent, the authors succeeded in their aim of bringing public ridicule down on at least one of the fields they targeted.

Whether they succeeded in showing that standards are low or that peer review is distorted by ideology in these fields is a difficult question. Lagerspetz (2020) notes that (with the exception of papers reporting – fabricated – data) submissions were more likely to be accepted by journals with lower impact factors than higher, perhaps indicating that any problem is not with the fields targeted, but confined to more marginal journals within them. He also notes that the authors seem to have learned from their rejections and reviews. All their initial submissions were desk rejected: it is only by rewriting papers in the light of feedback they were able to have some success. Perhaps the authors raised the genuine quality of the papers in the process. All accepted papers were withdrawn when the experiment ended, and those published were retracted, but perhaps some of them were genuine contributions to the fields, regardless of authorial intent.

These questions, interesting though they are, are not my questions. Rather, I’m concerned with the betrayal of trust involved in submitting a hoax paper to a reputable journal. I don’t think I am taking sides in describing what Pluckrose, Lindsay and Boghossian did as betraying reviewers’ and editors’ trust. While trust is a moral good, and its betrayal a pro tanto wrong, such betrayal is surely sometimes appropriate. If, for instance, someone trusts us to keep secret his plan to extort money from vulnerable people, we would normally have an obligation to betray his trust; conversely, someone’s being trustworthy in this kind of context is strong evidence they’re not a good person (Jones 2017). Pointing out that Pluckrose, Lindsay and Boghossian betrayed the trust that reviewers and editors placed in them isn’t question begging; it leaves open whether they were right to do so.^2^

Let me turn, now, to a very different instance of betrayal of trust in a (semi) academic context: social media. Social media is now a central medium for the communication of distinctively academic content: both scholarly discussion and information about academic institutions and the broader profession (Carrigan 2019). Academics on Twitter report that it has allowed them to find new collaborators, share ideas far more widely, discover new work and raise the quality of their own, and to support colleagues (Britton, Jackson, and Wade 2019). In the past 12 months, with travel difficult or impossible, it has become even more central to our lives as academics: it is now the primary means by which most of us communicate with fellow academics beyond our own department. Even prior to 2020, social media had become the most important news source for recent developments in educational institutions; not only in virtue of the tweets and updates of official sources and media organizations, but especially in virtue of how social media pools the collective wisdom of thousands of people employed in such institutions. Their first-hand reports provide us with the most up to date and detailed information concerning how our fields and our professions are evolving and the challenges we face. The second instance of the betrayal of trust occurred in this kind of context.

In July 2020, a neuroscientist and #metoo activist, BethAnn McLaughlin, announced on Twitter that an anthropology professor who was herself outspoken on issues of gender and race had died of COVID-19. That professor had remained anonymous, due the fact that she lacked tenure and was therefore vulnerable; McLaughlin was among the few people who knew her real identity. The death of @sciencing_bi caused shock and anger, directed in particular at Arizona State University: @sciencing_bi had told her followers that she’d been made to teach in person classes at ASU despite the virus. She attributed her illness to those classes. Almost immediately, however, the story began to unravel. Under pressure, McLaughlin admitted that she had fabricated @sciencing_bi (Bromwich and Marcus 2020).

Most of the people who interacted with @sciencing_bi had trusted her, in the following (minimal) sense: they had acquired beliefs on the basis of her testimony. Of course, they were not undiscriminating in accepting her claims. She took sides in controversies, and many people disagreed with some of her stances. But few people doubted her more mundane claims, asserted or implicated: I am a professor of anthropology; I work at ASU; I am of Hopi descent; my university requires me to teach in-person classes. They took her to be sincere in her utterances even those they might have disagreed with.

Once @sciencing_bi was revealed to be a sockpuppet – a fake account, set up by someone for the purpose of amplifying or supporting them online – some of those who were taken in acknowledged that there had been red flags all along, such as the fact that queries put to McLaughlin might be answered by @sciencing_bi and vice-versa. Moreover, a number of people had long had doubts about McLaughlin herself; she had faced accusations of bullying and marginalizing younger scientists and women of color in MeTooSTEM, the organisation she had founded. In light of these red flags, some of those who had been taken in blamed themselves for their credulity. As Michael Eisen, a professor of genetics at UC Berkeley put it, ‘I don’t want to stop being trusting, but even so, we should have known’ (Cara 2020). Eisen published a long Twitter thread, apologizing for his role in amplifying @sciencing_bi and not supporting those women who had expressed doubts about McLaughlin (Eisen 2020a).

We now have before us two examples of trust betrayed in (broadly) academic contexts. One, the case of @sciencing_bi, clearly involves malfeasance. The other, the so-called ‘grievance studies’ hoax, is harder to assess: some instances of betrayal in contexts like these are clearly permissible, and some are not.^3^ No matter how we assess the permissibility of the hoax, however, we might think that reviewers should have been more sceptical with regard to these papers (indeed, we might think, with Pluckrose, Linsday and Boghossian, that they should be more sceptical with regard to *all* the submissions they receive, applying higher standards to their claims). Similarly, we may think that the case of @sciencing_bi shows that we ought to be less trusting on Twitter. We might agree with Michael Eisen: ‘we should have known’.

Eisen and those who agree with him might think that these cases show that we have not been epistemically responsible. The epistemically responsible agent would have seen through @sciencing_bi. In this paper, I will argue against this view. I will argue we ought to continue to be trusting in contexts like these; we ought to continue to take people more or less at face value. I will argue that the epistemic benefits that flow from trust are too important, and too easily damaged, for us to risk becoming less trusting. We stand to lose more than we would gain. Given that academic networks are valuable very importantly (though not exclusively) for their epistemic benefits, becoming less trusting would severely undermine their value. We *shouldn’t* have known: *we* behaved epistemically appropriately.^4^ The lessons I will attempt to draw are quite general; that is, they’re intended to apply across a range of academic contexts. The two case studies I examine have a distinctive feature, however: they involved the authors passing themselves off as members of historically disadvantaged communities. This distinctive feature involves a distinctive cost of becoming less trusting in contexts like these: an increase in testimonial injustice.

## The Epistemic value of trust

Much – perhaps most – of what we know, we know on the basis of testimony, and (except in some unusual cases) we acquire knowledge through testimony only when we trust speakers. We trust that they are sincere and competent to testify. This is true in ordinary life: we acquire knowledge from passers-by, from friends and acquaintances, from news reports and from signs. We can do so only because we trust they are minimally sincere and competent. This is just as true in academic contexts, such as the context of reading a journal article or reading philosophy Twitter. We may reject the conclusions of a paper we’re reviewing, for instance, but we acquire knowledge from it nevertheless. For example, we acquire knowledge of the author’s position, and of the views of those people she cites.

Of course, that kind of knowledge is relatively trivial. We hope for more: we hope to acquire knowledge of significant truths. It’s important, to note, though, that the acquisition of knowledge of significant truths is dependent on the acquisition of knowledge of these relatively trivial facts; trust is therefore essential to the acquisition of the deepest truths. Consider peer review, which plays a central role in the generation and transmission of knowledge. When it works well, peer review filters out bad or unreliable work and improves the papers which pass through it.^5^ Peer review makes its contribution to knowledge production as a component in a much broader system of distributed cognition. The components of this system compensate for the limitations of individual agents: given my aptitude and the constraints on my time and resources, there are many things *I* don’t know, but which *you* might. Peer review and other cooperative and conflictual ways of distributing epistemic labor pool our knowledge. Together, we can collect data, analyse it and test hypotheses in ways that are out of reach for each of us alone. What we can do together is far more than the sum of what we can do apart: many questions are inaccessible to individual investigation, even aggregated individual investigation.

Cooperative and conflictual distributed cognition is essential to the generation and the transmission of knowledge. This is clearest in the sciences. Three and half decades ago, Hardwig (1985) pointed out that papers in physics might have as many as 99 authors. Since then, the number of authors has risen across many other disciplines as well, and may often number well into the hundreds (Mallapaty 2018). The paper reporting the detection of the Higgs boson had more than 5000 authors (Castelvecchi 2015). Groups of scientists working together in the same lab or across multiple labs bring a range of different kinds of expertise and different skills to bear on a common problem. Peer review and post publication review iterate the process, allowing multiple individuals, with different skills, backgrounds and biases to subject a paper to critical scrutiny. The very existence of science as a successful epistemic enterprise may owe more to the institutions that distribute epistemic labor than to the so-called scientific method.

Crucially, *trust* in the contribution that individuals and research groups make to the scientific enterprise is central to its success. No individual contributor to a scientific paper is in a position to verify the claims made by all the others, due to lack of time and of capacity. Were the methods implemented as described? Was the data analysed appropriately? Are the statistics reported accurately? While it is often possible to seek evidence sufficient to justify confidence in an answer to these questions, for lack of time scientists rarely do. Only if something strikes them as suspicious or somehow ‘off’ do they engage in such activities. Routine checking wouldn’t be a good use of their time: time spent on verification would be time they *don’t* spend on building on or testing the work they trust has been accurately reported. In fact, no scientist *could* check more than a tiny minority of the work they rely on even were they to do nothing else: there simply isn’t enough time.

Trust is therefore a practical necessity. It’s also required *in principle*: scientists are routinely unable to verify the reliability of all the tools they employ. They often lack the specialist knowledge, because they use tools (mathematical or physical) developed by specialists in other fields. In fact, there are cases in which – because methods and models have evolved over time – *no one* is in a position to verify them for themselves (Winsberg 2018). In these cases, the models may be opaque to all those who rely on them. Trust is necessary at every stage of the peer review process: trust in co-authors, in methods and tools, trust that the authors of the paper we review are sincere and minimally competent in the appropriate sphere, trust in editors and other reviewers. Without trust, we could not generate and transmit knowledge though the journal system.

While trust is essential to the generation and transmission of knowledge in the sciences, it is less obvious that it is anywhere near as central in the fields that the ‘grievance studies’ hoaxer targeted, or in the humanities more generally.^6^ It’s surely the case that we need to trust others to different degrees in different fields, because the balance of claims that readers are able and expected to verify for themselves versus those that must be taken on trust varies from field to field. Fleisher (2020) helpfully distinguishes between two kinds of claims a philosopher might make in a paper (or, indeed, in a seminar or an interview): *advocacy role* and *evidential role* claims. Advocacy role claims are those for which arguments are offered, whereas evidential role claims are those asserted without argument. With regard to these latter claims – e.g. the claim that Mencius worked in the Confucian tradition of philosophy, or indeed that Fleisher distinguishes between evidential and advocacy role claims in the manner I have just suggested – trust is essential. Whereas we’re expected to assess the plausibility of an argument on the basis of the evidence provided, we’re expected to accept evidential role claims on the say-so of the author.

While Fleisher’s focus is philosophy, the distinction is a helpful one for illuminating the different extents to which different fields require us to take claims on trust. No field of inquiry asks us to take every significant claim on trust, but some rely on evidential role claims more than others. Mathematics and formal logic represent one extreme: almost every claim in a professional publication in these fields is in principle verifiable by readers without the need for cross-checking. Even here, however, trust is expected of most readers: proofs can run to many pages, and only those with a special interest in precisely *that* problem will attempt to check every line. Others may be content to understand the overall logic of a proof, or simply take it on trust (that is, on the word of reviewers, pre- and post-publication) that a problem has been solved. At the other end of the scale, historians may (often) be more concerned with reporting findings from the archives than with offering new interpretations of existing evidence; the first must be take on trust to a far greater extent than the second (archives may be inaccessible or too vast for practical verification even for most specialists). The balance of advocacy and evidential role claims may vary even across subfields. For example, some work in sociology is largely devoted to the reporting of qualitative data that would be difficult for readers to verify, with (more easily verifiable) interpretations of that data playing a smaller role; other work in sociology relies on publicly accessible quantitative data sets that are accessible by anyone, such that only time and expertise limits our capacity to verify the work; still other work offers theoretical speculations that are, in principle, entirely open to assessment by readers.

To the extent to which arguments are offered for claims – the extent to which a paper or a (sub)field relies on advocacy role claims – we’re able and sometimes expected to accept them only when we find them persuasive. The degree to which such claims are central varies from field to field, and even within fields. Science, with its heavy emphasis on data, therefore tends to rely more on trust than does philosophy. But such contrasts should not be overblown. Science, too, puts forward arguments: *that these data support this hypothesis*, for instance. And philosophy is heavily reliant on evidential role claims, which may be more or less difficult to check (think of an interpretation of an inaccessible text). Even those of us who are experts in a particular field must continue to trust others who contribute to that field: they often bring slightly different ranges of skills and background knowledge, and their evidential role claims play an essential role in constructing their arguments. Trust in one another remains indispensable, even in the humanities, for knowledge production to continue.^7^

Perhaps surprisingly, academic Twitter is also an important source of knowledge. Much of what ‘we’ know (for a variety of values of ‘we’: members of our profession; fellow academics; those with an interest in higher education) we know as a consequence of the way in which knowledge is shared and pooled on social media. We often know about our *own* institution via these kinds of routes: many academic institutions are simply too big for individuals to get a good sense of developments and conditions through face to face interactions. The people we meet with every day may be quite unrepresentative (many academics with tenure or on the tenure track would have little idea of what it’s like to be an adjunct were it not for testimony on Twitter). In particular, we rely on social media to learn about those aspects of our institution that the official stories would rather not have highlighted.

Similar points hold true for higher education more generally, across whatever nation we live in and across the world. We know that many of the problems our institution confronts are widespread (again, adjuntification is a good example) and we may hope to learn how they can be confronted. Importantly, most of the people whose knowledge Twitter pools are individuals we have never met offline. They live and work across the country and across the world. Granted, we can sometimes be confident that they are who they say they are, because they are embedded in networks of people, some of whom we *have* met. But often the network contains few people we have met and who are also in a good position to vouch for the identity of particular members. As the case of @sciencing_bi shows, such (apparent) verification is itself sometimes deceptive.

Again, we often could take steps to verify the existence of our Twitter interlocutors: we can google, send emails to their contacts, and so on. But we can’t be expected to check more than a small proportion of those we engage with: the opportunity costs of such an investment of time are prohibitive. Moreover, some people (like @sciencing_bi, were she real) have good reasons to dissimulate their identity online, because their employers or powerful people might retaliate against them, or because they need to keep their professional and Twitter identities separate. If we are to acquire knowledge from Twitter, we’ll be forced to trust.

## The nature and fragility of trust

Standardly, trust is analysed as reliance plus something extra (Hawley 2014). To rely on someone or something is to figure it into our plans (Holton 1994). We plan on its occurring, rather than make contingency plans in case it fails. We usually rely on the reliable: I might rely on Aisha to pick me up from the airport in part because I believe she won’t fail to do so, and Aisha may rely on her car to get her there because it never lets her down, but when our options are bad we may rely on the unreliable (Dormandy 2020). A climber might rely on a frayed rope to bear her weight as she navigates a tricky crevasse, because her other options are worse. But the climber who relies on an unreliable rope doesn’t count as trusting it: reliance isn’t sufficient for trust.

I won’t attempt to adjudicate between various account of what must be added to reliance to yield trust. Whatever trust consists in, we can identify instances of it by the fact that the trustor takes the reactive attitudes to the trustee (Holton 1994). To test whether an agent trusts or merely relies on a person, we only need to ask how she would feel if the trustee let her down. If Aisha’s formerly reliable car won’t start, she will feel disappointed but not *betrayed*.^8^ Similarly, if I am forced to rely on feckless JoJo to pick me up, I won’t feel betrayed by his failure. I didn’t trust him enough for that.

We’ve seen that it’s possible to rely on someone or something without believing they are reliable. It’s widely held that it is also possible to trust someone without believing they are trustworthy; that is, when we’re not confident that they won’t let us down. First, some argue, trust can be voluntary (Holton 1994): I may decide to trust someone somewhat independently of what I believe about them. Second, trust may be ‘therapeutic’ (McGeer 2008). That is, one agent may trust in the hope (but not the expectation) that the experience of being trusted will have an educative or formative effect on the trustee. Thus, parents might trust their daughter to behave responsibly while they’re out of town, even though they fully expect her to hold a party instead.

I think this is a mistake (see Hieronymi 2008; Keren 2014): trust requires belief (Hawley 2014), and belief isn’t voluntary. It is certainly possible to decide to rely on someone when we don’t believe that they will come through (even when we disbelieve that they will come through): we simply need to set aside our doubts and plan on them coming through. We can also entrust something to such people. But reliance and entrusting both fall short of genuine trust. Only when those we have trusted let us down do we feel *betrayal* (rather than disappointment, or something of that kind). The feeling distinctive of betrayal is a response to an unexpected let down; we feel only disappointment or sadness when the let down is expected or not unexpected.

Trust therefore tends to expose us to greater harms than reliance, in two ways. First, it exposes us to the harm of betrayal, not mere disappointment. Second, we prefer to rely and we tend to rely more on the trustworthy, because we are much more confident that they will take our interests into account: we therefore tend to have more to lose when we rely on the trustworthy than on the merely reliable. When exposed to a risk of serious harm, rational agents take precautions. They either ensure that risks are minimized or they hedge against loss. For example, the rational investor may place some of her money in assets she (carefully) assesses as safe, and ensure that only those she can afford to lose are invested in riskier assets. She scrutinizes potential investments to assess risk and plans for failure. The paradox of trust is that it exposes us to serious harms, which makes steps of these kinds rational, but it is destroyed if we take them.

Trust is ‘a fragile plant’, one that may not survive ‘inspection of its roots’, even if it was healthy prior to such reflection (Baier 1986, 260). ‘Trust but verify’ is oxymoronic: if you tell your friend you trust her to look after your kids but install security cameras to check up, she may rightly protest that you don’t trust her at all. If you make contingency plans for her letting you down, you don’t (fully) trust her. The trustor refrains from *epistemic* precautions too. Trust is constitutively opposed or unresponsive to evidence (Jones 1996; Hieronymi 2008). As Keren (2020, 120) puts it, trust entails ‘insensitivity to evidence’; once we’ve decided to trust someone, we won’t seek evidence for the trusted person’s reliability, nor even deliberate on available evidence.

We are able to trust one another, in part, because most people are trustworthy. They are trustworthy not only in the sense that they are reliable and honest, but also and especially because they respond positively to being trusted; they are, in Jones words, ‘directly and favorably moved by the thought that we are counting on her’ (Jones 1996, 4). We can and should do more to make ourselves trustworthy in response to the trust that others show in us (Almassi 2012; O’Neill 2020). But it would be naïve to think that everyone will be moved by the thought that others are relying on them, and if people trusted undiscriminatingly they would be widely exploited by the unscrupulous, and the benefits of trusting would be lost. There is extensive evidence that ordinary agents are much less reliable at detecting deception than we take ourselves to be (Michaelian 2010; Shieber 2012), but the fact that trust survives and is widespread is very good reason to be confident that it is not undiscriminating. In fact, we always exercise ‘epistemic vigilance’ (Mascaro and Sperber 2009; Sperber et al. 2010; Harris 2012), and we are typically reliable enough at filtering out bad testimony to avoid exploitation (Mercier 2017). In low-stakes situations, we tend to be very trusting, but we are more vigilant when more is at stake, and especially when the speaker may have an interest in deceiving us (Mercier 2020). We vary our standards for trust depending on the speaker and the situation. Most of us have learned to be extremely sceptical of unsolicited offers from Nigerian princes and of requests for articles from journals we’ve never heard of.^9^

How do we succeed in preserving trust, while nevertheless protecting ourselves against fakes and frauds? Epistemic vigilance needs to be *implicit* if trust is to survive. While trust is destroyed by explicit vigilance, it is compatible with, and can flourish, in the face of implicit mechanisms that monitor for warning signs. As Elizabeth Fricker suggests, the kind of active monitoring of competence and sincerity required to turn testimony into knowledge is ‘sub-personal’ (Fricker 1994). Explicit monitoring (which corrodes trust) begins only when implicit mechanism (which leave it intact) sound an alarm.

The apparent paradox of trust is therefore easily resolved: while trust is incompatible with explicit vigilance, we can and do limit our exposure to risk when we trust by engaging in implicit monitoring of speakers for evidence of insincerity, ill will or incompetence. We’re now also in a better position to consider the suggestion that we ought to trust less in contexts like our two case studies: both to set out what a reduction in trust might consist in, and to set out the stakes and risks of such a reduction. We can trust less either by increasing our implicit vigilance or by calibrating our level of explicit vigilance. In the next section, I’ll assess the prospects for these two kinds of vigilance, in light of the risks that stem from the fragility of trust. Can we succeed in increasing our vigilance without destroying trust, and thereby losing the goods it brings us?

## Trusting less

Let’s consider, first, the suggestion that we should increase our implicit vigilance. Since implicit vigilance does not seem to threaten trust, this seems the safest strategy we might adopt. Granted, implementing this strategy is easier said than done. It’s no simple matter to change the sensitivity of implicit mechanisms. Still, it’s quite probable that we could raise these standards. We might make ourselves more sensitive to potential problems in the papers we review, for instance, by reading the near-daily posts at *Retraction Watch*, many of which concern papers retracted due to questionable research practices and even outright fraud.^10^ We might google for the many incidents of catfishing and the like online, and thereby make ourselves more sensitive to potential problems on social media. By engaging in these kinds of activities, we may well succeed in changing the sensitivity of the relevant implicit mechanisms.

While intentional reconfiguring of these mechanisms is no doubt possible, intentionally engaging in these kinds of steps is likely also to reconfigure our explicit vigilance. Read *Retraction Watch* every day and every time you review a paper you’re likely to be on the lookout for problems. Sensitize yourself to the potential for deception on social media and you’re unlikely to be able to turn off the awareness as you engage online. Intentional behavior like this is of course explicit behavior and its effects are at least as much on our explicit vigilance as on our implicit. While implicit vigilance need not threaten trust, we’ll have great difficulty altering its sensitivity without also altering explicit vigilance (it’s noteworthy, additionally, that on dual process models of cognition, the function of implicit vigilance is to make us aware of problems, so even if we succeeded in making ourselves implicitly but not explicitly more vigilant, the same problems will tend to arise: we will still engage in explicit assessment of trustworthiness, and such explicit assessment tends to lead to distrust, I will argue).

If we are to succeed in preserving trust while also becoming more vigilant, trust will need to survive an increase in *explicit* vigilance. This is true whether we think the best way to reconfigure our trust is by altering implicit mechanisms (and thereby also altering explicit) or whether we think it is better to focus on explicit mechanisms more directly. There is, surely, grounds for optimism here. Trust is not all or nothing. We can surely hope to become slightly less trusting without being *dis*trusting, and thereby without undermining the epistemic value of our trusting relationships. I will suggest that though trust comes in degrees, explicit trust tends toward being binary: I will argue that it is very difficult to make ourselves slightly less trusting, and attempts to do so will tend to cause us to become distrustful.

Why should trust, which clearly comes in degrees, tend to toward being binary when we attempt to recalibrate it? The central reason is that evidence of (un)trustworthiness is rarely unequivocal: ‘typically the evidence we find under-determines judgments of trustworthiness or lack of trustworthiness’ (O’Neill 2020, 19). Assessing trustworthiness (to the limited extent we’re able to do so at all) is an exercise in hermeneutics, and the interpretive frame is liable to play a major role in assessment. Because that is the case, it is inherently difficult intentionally to recalibrate explicit trust in a fine-grained manner. Instead, measures that aim at making ourselves very slightly less trusting are likely to cascade into the wholesale destruction of trust. A glancing inspection of the roots of the fragile plant that is trust can kill it, as Baier pointed out, and though she developed a test for when trust is justified, she also noted that it may be ‘the better part of wisdom ….not to use it except where some distrust already exists’ (Baier 1986, 260).

Cascades in interpretive framework are liable to occur because when we have our suspicions raised, we will likely see reasons for doubt everywhere. Because the evidence is inherently ambiguous, evidence that previously might have been understood as demonstrating trustworthiness is likely to appear as showing just the opposite. His friendliness, previously evidence of his warmth and care, is now seen as manipulative; his interest in us now appears a little creepy. Ordinary interactions are full of signs that we would interpret as red flags were we looking for them. Is the speaker’s nervousness or gaze aversion shyness, or rather a sign she’s deceptive? Or on the contrary, is she too confident, too brash? Perhaps she’s overcompensating. Perhaps her confidence stems from a privileged upbringing (not the background she claims for herself)? Is *that* really a phrase that someone from her (supposed) background would use? We might notice inconsistencies in her story – perhaps stemming from memory failures – and attribute them to deception. When we’re on the lookout for signs of untrustworthiness, evidence of such signs becomes salient, and evidence inconsistent with it becomes pallid. Confirmation bias (Nickerson 1998) kicks in, with each piece of evidence that might indicate a lack of trustworthiness making us more sensitive to such signs and more liable to find them in the inherently ambiguous evidence. What began as an attempt to become just a little less trusting rapidly cascades into the conviction that the person is unreliable.

Consider the babysitting example again. We saw that installing security cameras would draw justified protests: *I thought you trusted me!* But there are few actions we could take that wouldn’t demonstrate distrust. Even just rethinking our history of past interactions with our friend, to see whether they might indicate anything off would tend to undermine trust, by sensitizing us to the ways in which our inherently ambiguous evidence could support distrust. We might easily be led to reinterpret past incidents – say what we then saw as friendly teasing – as instead indicating something more sinister.

The inherent ambiguousness of the evidence, and the dramatic difference that interpretive frame makes to it, is well illustrated by cases in which trust really is betrayed. Consider @sciencing_bi again. Michael Eisen now says that he and others ‘should have known’ that she wasn’t who or what she appeared. There were, he points out, red flags all along: ‘in retrospect, the signs that this was all bullshit were there’ (Eisen 2020b). But what Eisen sees as red flags *now* are no such thing from within a more trusting interpretive frame. The evidence we now see as showing that @sciencing_bi was not who she said she was is all consistent with, and arguably equal probable, were her story true.

@sciencing_bi used stock photos to illustrate posts about her own activities; she provided BethAnn McLaughlin’s Venmo account when asking for donations; she’d provided a link to an online document that was connected to McLaughlin’s Google account (Aldhous 2020) she sometimes answered questions addressed to McLaughlin and vice-versa. In retrospect, all of this can be seen to constitute evidence that @sciencing_bi and McLaughlin were identical. But seen from a trusting stance, they don’t look much like red flags at all. There are many reasons why someone might use stock photos, ranging from laziness to an attempt to throw those who might identify her off the track (given her supposed vulnerability). Similarly, the use of McLaughlin as a proxy might be a smart move to hide her real identity (since McLaughlin claimed to be in a relationship with @sciencing_bi, the choice of her is easily explicable; this same fact might explain why one would sometimes answer questions addressed to the other).

When we test the hypothesis *that @sciencing_bi is suspect in some way*, we’ll find plenty of evidence to support our hypothesis. Equally, though, many actually trustworthy speakers would fail that test. Inconsistencies are common; apparently suspect word choices and the many cues that we commonly take to indicate lack of sincerity (e.g. not meeting our gaze) are routine and highly unreliable as cues to deception; in fact, deceivers hijack the cues widely regarded as indicating trustworthiness quite well (see Shieber 2020 for review). Raise our explicit standards for trusting, and we will likely filter out more good cases than catch bad (given the base rates). As a consequence, our capacity to acquire knowledge from testimony will be severely impaired. We must adopt a trusting interpretive frame to acquire knowledge via testimony, and within that frame @sciencing_bi will seem trustworthy. It’s very unlikely we’ll succeed in calibrating our trust so that she would be filtered out while our capacity to acquire knowledge from testimony isn’t severely damaged.

The same sort of lesson applies to trust in other contexts, such as in the explicit assertions and implied claims made by the authors of journal articles. Journal articles (especially in some disciplines) tend to take a more standardized form, so there may be fewer ambiguous stylistic cues to interpret. But being vigilant for hoaxes risks making us distrustful of papers that make surprising, or politically or morally controversial, claims. It is these kinds of claims that are especially likely to be put forward in hoax papers (to demonstrate low standards or bias). If we’re on the look-out for hoaxes when we assess such papers, we run a significant risk of interpreting the *content*, as well as the wording, as evidence of deceit. In the humanities, where there is more tolerance of stylistic variety, this is especially likely to be a problem.

## Trust and testimonial injustice

The discussion to this point has been pitched at a level of generality: it has concerned the possibility and the costs of recalibrating trust levels in academic contexts (broadly construed), without any attempt to distinguish different kinds of contexts. But the examples with which we started have features that other betrayals of trust lack. In this section, I will suggest that these features make any attempt to recalibrate trust more costly than they might be in other contexts.

The ‘grievance studies’ hoax and the case of @sciencing_bi are very different from the Sokal hoax and other academic impostures inasmuch as these cases involve individuals attempting to pass themselves off as members of historically disadvantaged groups.^11^ This kind of fraud seems to be increasingly common on social media. A recent example involved a white male chemistry professor tweeting under the name ‘The Science Femme, Woman in STEM’, and claiming to be an immigrant woman of color (Flaherty 2020). These frauds threaten a kind and degree of harm that others do not. They threaten to exacerbate the testimonial injustice to which minorities are already subject, with harms accruing both to them and to all the fields in which their voices are sorely needed.

An agent suffers testimonial injustice when lower credibility is placed in her assertions due to identity prejudice on the behalf of hearers (Fricker 2007). Thus, if she is less likely to be believed because she’s a woman (or Black, or trans, and so on) she suffers testimonial injustice. There is good reason to suspect that such identity prejudices are not confined to those who endorse them. A feminist may harbor such a prejudice against women; someone sincerely committed to racial equality may harbor prejudices against Black speakers. Since we may know or suspect we have such prejudices, we may know or suspect we tend to give less credibility to some speakers than they deserve.

When (say) white cis males attempt to pass themselves off as members of historically disadvantaged groups on Twitter, they attempt to *inflate* the credibility of their testimony. How can this be, if such groups are subject to testimonial injustice? There are at least three reasons why such testimony may be given more credibility than the same testimony from these individuals posting under their own names. First, we accord people a great deal of credibility when they testify about their own experiences; perhaps because we think that the quality of information and its reliability degrades the more links there are in a chain of transmission. That allows a white person who wants to make claims about racism to boost their credibility by speaking in the guise of someone who has been its victim; that same person reporting the experiences of others has less credibility. Second, speakers receive a credibility boost when they give testimony that is perceived as contrary to their own interests (Berinsky 2017), perhaps because such testimony is taken as a sign of scrupulous honesty; that allows those who want to maintain that racism is greatly exaggerated to enhance their credibility by posing as a member of a group that can be expected to have experienced racism. Third, sympathetic people who are aware of the reality of testimonial injustice and of implicit prejudice might attempt to compensate for it, either by deliberately boosting their credibility assessments or through habituation. Attempting to recalibrate levels of trust so as to avoid such hoaxes threatens these kinds of efforts.

In the long-term, we may hope to eliminate the identity-prejudices that cause testimonial injustice. But justice demands we act *now*, to minimize harm to those who are its victims. Fricker recommends that we become more self-reflexive: when we might be subject to identity prejudice – i.e. when our interlocutor is a member of a group with regard to which we know that we or people like us tend to be prejudiced – we should check to see whether prejudice has had an impact on this occasion. The virtuous agent ‘should shift intellectual gear out of spontaneous, unreflective mode and into active critical reflection in order to identify how far the suspected prejudice has influenced her judgment. If she finds that the low credibility judgment she has made of a speaker is due in part to prejudice, then she can correct this by revising the credibility upwards to compensate’ (Fricker 2007, 91). Rather than urging an increase in explicit vigilance, that is, she urges an increase in explicit trust. Such efforts do not run the risks I’ve argued for in the case of explicit vigilance (though they might run risks of their own). Her advice assumes, however, that introspection can reliably uncover signs of prejudice in us; the evidence seems to suggest that this is false (Levy 2017). That being the case, we may do better to inflate credibility assessments across the board when identity prejudice may be in play.

Such boosts seem to be inconsistent with the vigilance for red flags and other steps that seem required to make us less liable to be taken in by future hoaxes. Correcting for testimonial injustice in these kinds of contexts calls for *more* trust, not less. Being attentive to red flags will tend to make epistemic injustice worse. If we should be suspicious, it is toward those people to whom we might tend to accord an *excess* of credibility – members of dominant groups, who have a great deal of cultural capital (Medina 2013). We might, however, expect a rough correlation between apparent red flags and lack of cultural capital: the smoother one’s presentation and the more adept at presenting oneself as possessing the markers of expertise, the better one is likely to be at covering one’s tracks.

Other academic hoaxes may threaten the credibility of work in particular fields. Sokal aimed at deflating the credibility of work in cultural studies and Pluckrose, Lindsay and Boghossian aimed at decreasing the credibility of ‘grievance studies’. Hoaxes that involve passing oneself as a member of a disadvantaged group often threaten work in particular fields too (The Science Femme explicitly made common cause with Pluckrose, Lindsay and Boghossian) but they also threaten the credibility of members of the groups they pretend to belong to. They thereby undermine their ability to contribute to many fields, especially those in which they remain a minority. The harm of such hoaxes is accordingly greater. But we may need to tolerate these harms, since attempts to correct for them by recalibrating trust may make things worse rather than better.

## Conclusion

Trust is epistemically valuable (perhaps it is also intrinsically valuable). It is epistemically valuable because trust is a necessary condition of the acquisition of knowledge via testimony, and testimony is a principal source of our most significant knowledge. We would prefer to trust all and only the trustworthy, but such perfection is not available: we must rely on cues for trustworthiness, and these cues can lead us astray (not least because the untrustworthy can mimic them). As a consequence, our trust is sometimes betrayed, as it was by Pluckrose, Lindsay and Boghossian, and by @sciencing_bi. We might reduce our vulnerability to such betrayals by raising our epistemic standards; being on the lookout, to a greater extent than we now are, for evidence of untrustworthiness. I’ve suggested that if we do so, we’ll likely find ourselves distrusting much more often than we now do, and many of those we distrust will in fact be trustworthy. While it is arguable that a small increment in our threshold for trusting might not lead to a significant rise in false positives, such a small increment is unlikely to be achieved. Raise our threshold for trusting, and we are likely to filter out too many trustworthy informants, thereby losing the opportunity for acquiring significant knowledge. We will also subject more informants to testimonial injustice.

There’s an obvious objection lurking in the wings. Continuing to trust at our current levels leaves us vulnerable. Of course (as we’ve seen), vulnerability is inherent to trust, but while falling for the occasional hoax or scam is an acceptable price to pay for the epistemic goods we currently reap from academic networks, this is true only so long as fraudsters remain at a low level. If we’re too trusting, we can expect a rise of such scams, and soon our epistemic networks will cease to function as sources of knowledge. We must be able to calibrate trust to the actual likelihood of encounters with the trustworthy, or our epistemic networks will fail. Conversely, the fact that these networks have survived the rise of the predatory journals (more or less) intact shows that we *do* engage in such calibration. Most of us have learned to be sceptical of emails that solicit our contribution to a special issue that needs just one more paper.

There’s a fairly obvious reply to this obvious objection, though. Our levels of implicit vigilance are calibrated by feedback, and we become more vigilant as levels of fraud rises. We don’t need to adopt policies of explicit vigilance to prevent fraud. In well-functioning epistemic networks, we can expect deception to be maintained at low levels through automatic adjustment of vigilance to detect it. Our networks continue to function only if they have in place mechanisms for detection of deception. Academic networks have many such mechanism, distributed across multiple agents and individuals (that’s how these cases tend to come to light). We rely on what Grasswick (2020, 177) describes as ‘a vast array of already functioning social practices that embody networks of epistemic trust in inquiry and testimony’ to ensure that we can function as epistemic agents. We must trust in them, as well as one another.^12^

## Notes

1. In assessing whether the hoax really revealed low standards in the fields targeted, what matters is not the number of *papers* but (inter alia) the number of unique submissions, where a unique submission is a paper/journal pair. The project featured 48 unique submissions. The team ‘retired’ six papers as unlikely to be accepted and rewrote others in the light of feedback after rejection (this information is drawn from their project factsheet; https://drive.google.com/drive/folders/19tBy_fVlYIHTxxjuVMFxh4pqLHM_en18). All their initial submissions were desk rejected; it was only after they had adjusted the papers that they began to have some success. These data clearly indicate that journals in the fields targeted will not publish just anything that comports with their ideologies; they are, however, compatible with these journals using irrelevant or low standards.

2. A reviewer for this journal suggests that this hoax may not have involved a betrayal of trust at all. The reviewer suggests that we wouldn’t ordinarily describe a quality control inspector who went undercover to see whether a factory was abiding by appropriate protocols as betraying our trust. The reviewer is surely right that the quality control case is not clearly a betrayal of trust: whether or not it is depends on further details. If the inspector works on the line and establishes friendly relations with others, in order to pump them for information, I think we’d have no hesitation in describing her as betraying the workers’ trust. Were she instead to simply walk through the factory unobtrusively, we wouldn’t see her as betraying anyone’s trust. The grievance studies hoax falls somewhere in between these two kinds of cases. Unlike the unobtrusive inspector, it involves unsuspecting individuals relying on the hoaxers (to accurately report data and sincerely report arguments, to represent themselves truthfully and fairly). Insofar as it is appropriate to say that we (in our capacity as editors and reviewers) trust authors to be truthful, it seems appropriate to see Pluckrose, Lindsay and Boghossian as betraying our trust.

3. There have been many such hoaxes previously. The best-known is the Sokal hoax (see, e.g. Hilgartner 1997), in which a physicist deceived a cultural studies journal into publishing an article arguing that key concepts in physics were social constructs. More recently, such stings have been aimed mainly at predatory journals, which exercise little or no quality control so long as authors commit to paying a publication fee. For instance, an Australian computer scientist had a paper called ‘Get me off Your Fucking Mailing List’ accepted for publication by one such journal (Safi 2014) and a sting operation by a journalist indicates the problem is widespread (Bohannon 2013). The Sokal hoax was of dubious legitimacy: it is neither surprising nor revelatory that a journal without peer review would publish an article by a respectable physicist that asserted that contemporary physics and contemporary postmodern theory are mutually supportive. The betrayal of the trust of the predatory publishers is easier to justify: almost everyone who works in some capacity for them knows they are scams and do not deserve to have their trust respected. Moreover, these journals are epistemic pollutants (Levy 2018); limiting their influence is a laudable goal.

4. Framing the discussion around epistemic responsibility brings out the ways in which the problem on which I focus here is closely parallel to what has come to be known as the extended knowledge dilemma (Clark 2015; see Smart and Clowes 2021 for recent discussion). On a narrow construal, the dilemma arises only for those who accept that the mind may literally extend beyond the skull, and stems from the apparent fact that the automatic endorsement required for an external resource to count as part of the mind seems inconsistent with the responsible deployment of the epistemic virtues. Those who reject the extended mind, in favor of cognitive embedding (e.g Rupert 2009) face much the same dilemma, albeit perhaps in an attenuated form, because responsible endorsement and smooth deployment of external cognitive resources will often be incompatible. Similarly, the trust that (I will argue) is required for securing the epistemic benefits of work in most branches of academic research might seem incompatible with the epistemic virtue of a conscientious scrutiny of claims. Responses to the extended knowledge dilemma have tended to focus on whether automatic endorsement is always required for cognitive extension; the solution put forward here, in effect, questions the value of the putative epistemic virtue instead (see Bishop 2000 for a parallel argument against responsibility in epistemology).

5. Too often, peer review does not work well, and there are serious proposals for it replacement (see, for instance, Heesen and Bright 2020). Such proposals are worth taking seriously; perhaps the vulnerability of the current system to hoaxes might provide further evidence in their favor. I set this issue aside for the purposes of this paper; I’m concerned with whether we should be more vigilant *in* peer review, rather than whether we should replace peer review. Further, whether or not the current journal system survives, trust will always remain central to well-ordered intellectual inquiry, and the issues that peer review raises in this regard will remain important.

6. I thank a referee for *Social Epistemology* for making the importance of this issue clear to me.

7. Cassam (2018) has suggested that whereas we must defer to scientists, all of us can and should assess the claims of those working in the humanities (or, at any rate, historians) for ourselves. He therefore commits himself to the view that advocacy role claims play a very much smaller in these arenas than in the sciences. Elsewhere, I’ve argued that Cassam seriously underplays the role of specialist expertise in the humanities, and therefore the extent to which non-experts must trust their claims to be able to acquire knowledge from reading them (Levy 2022).

8. Not normally, at any rate. Nguyen (forthcoming) argues that we may trust inanimate objects.

9. As a reviewer for this journal points out, the fact that scams continue to exist is good evidence that some people fall for them. There’s ongoing debate over the extent to which predatory journals deceive those who publish in them. Many of those who submit are aware that these journals have extremely low standards: they submit because their institutions provide incentives for publication, with little regard to quality (Xia et al. 2015; Demir 2018). Some report ignorance of the nature of the outlets; in some cases they are surely honest in doing so, though in others they may be seeking to excuse themselves. Since there are no incentives for handing over money or account details to financial scammers, we can be more confident that those who fall for them are deceived. But these scams may flourish even in the face of very high levels of mistrust. The cost of generating millions of scam emails is extremely small, and the potential pay-off for scammers high, so they need only to reel in a tiny proportion of the targeted population to reap a large return. Scams are lucrative if the scammers ensure that they don’t waste time on the sceptical. Herley (2012) argues that the (to us) obvious red flags these emails contain are design features: they filter out all but the most gullible recipients.

10. https://retractionwatch.com. It is important to emphasise that papers may be retracted (often by their authors) due to the detection of honest mistakes or due to an inability to reproduce the results. Having a paper retracted is not always a sign that a researcher is dishonest or incompetent; sometimes, it is in fact a marker of scrupulous honesty.

11. Interestingly, perhaps to forestall this criticism Pluckrose, Lindsay and Boghossian used a variety of names for their submissions, and only a small proportion of the names used signalled minority identity. Of course, in most fields names are revealed only to editors; given their subject matter, the papers may have been read (and may have been intended to be read) as voicing the thoughts of members of minority groups.

12. I am grateful to three reviewers for *Social Epistemology*, as well as the editor, for very helpful comments. Work leading up to the publication of this article was supported by the Wellcome Trust (WT 104848/Z/14/Z) and the Australian Research Council (DP180102384).
